# Chromosomal copy number and mutational status are required to authenticate ovarian cancer cell lines as appropriate cell models

**DOI:** 10.1007/s11033-024-09747-4

**Published:** 2024-06-28

**Authors:** Britta Stordal, Angela M. Farrelly, Bryan T. Hennessy

**Affiliations:** 1 Department of Natural Sciences, Middlesex University London, The Burroughs, Hendon, London, NW4 4BT UK; 2 Department of Medical Oncology, Beaumont Hospital and Royal College of Surgeons in Ireland, Dublin, Ireland

**Keywords:** BRCA1, Cisplatin, Chromosomal copy number, Ovarian cancer, Mutation, STR profiling

## Abstract

**Background:**

The mutational status of ovarian cancer cell line IGROV-1 is inconsistent across the literature, suggestive of multiple clonal populations of the cell line. IGROV-1 has previously been categorised as an inappropriate model for high-grade serous ovarian cancer.

**Methods:**

IGROV-1 cells were obtained from the Netherlands Cancer Institute (IGROV-1-NKI) and the MD Anderson Cancer Centre (IGROV-1-MDA). Cell lines were STR fingerprinted and had their chromosomal copy number analysed and BRCA1/2 genes sequenced. Mutation status of ovarian cancer-related genes were extracted from the literature.

**Results:**

The IGROV-1-NKI cell line has a tetraploid chromosomal profile. In contrast, the IGROV-1-MDA cell line has pseudo-normal chromosomes. The IGROV-1-NKI and IGROV-MDA are both STR matches (80.7% and 84.6%) to the original IGROV-1 cells isolated in 1985. However, IGROV-1-NKI and IGROV-1-MDA are not an STR match to each other (78.1%) indicating genetic drift. The *BRCA1* and *BRCA2* gene sequences are 100% identical between IGROV-1-MDA and IGROV-1-NKI, including a *BRCA1* heterozygous deleterious mutation. The IGROV-1-MDA cells are more resistant to cisplatin and olaparib than IGROV-1-NKI. IGROV-1 has a mutational profile consistent with both Type I (*PTEN, PIK3CA and ARID1A*) and Type II ovarian cancer (*BRCA1, TP53*) and is likely to be a Type II high-grade serous carcinoma of the SET (Solid, pseudo-Endometroid and Transitional cell carcinoma-like morphology) subtype.

**Conclusions:**

Routine testing of chromosomal copy number as well as the mutational status of ovarian cancer related genes should become the new standard alongside STR fingerprinting to ensure that ovarian cancer cell lines are appropriate models.

**Supplementary Information:**

The online version contains supplementary material available at 10.1007/s11033-024-09747-4.

## Introduction

Worldwide, there were 324,398 new cases of ovarian cancer in 2022, accounting for 1.6% of cancer cases [1]. The most common histological type of ovarian cancer is epithelial representing approximately 90% of all ovarian tumours [2]. Epithelial ovarian cancers frequently have a high amount of chromosomal instability. Increased total and regional chromosomal instability are associated with increased tumour grade by Broder’s classification, but not FIGO stage [3]. Within in each FIGO stage as tumour grade increases there is a decrease in the 5-year survival rate [2].

Epithelial ovarian cancers have been traditionally divided into two categories (Type I and Type II) corresponding to two main pathways of tumorigenesis [4]. Type I tumours arise in a stepwise manner from borderline tumours and include low-grade serous carcinomas, mucinous, endometroid and clear cell carcinomas [5]. Type I tumours are characterised by a higher percentage of either *KRAS, BRAF, PTEN, PIK3CA* and *ARID1A* mutations and a low proliferation index [4, 5]. Type II includes high-grade serous carcinoma, malignant mixed mesodermal tumours and undifferentiated carcinomas. Type II tumours are rarely associated with precursor tumours and it has been suggested that they develop de novo from surface epithelium or inclusion cysts of the ovary as well as within the fallopian tubes. Type II tumours are characterised by frequent *TP53* mutations (50–80%), *BRCA1/2* mutation or methylation, a high proliferation index and increased chromosomal instability [4, 5]. Patients with Type II tumours have a worse disease-free survival [6] and disease-specific survival [7] compared to Type I.

Classifying ovarian cancer into Type I and Type II like any dichotomous classification system is useful but is simplistic and requires additional sub-branches. High-grade serous carcinoma (HGSOC) (Type II) and low-grade serous carcinomas (Type I) best fit into a dichotomous classification, with different precursors, and distinct molecular profiles [8]. Type I tumours are not homogenous, even within the histological types, and can have poor clinical outcomes [8] For example, ovarian clear cell carcinoma can be divided into subtypes through gene-expression clustering with differing progression-free survival.

Similarly, gene-expression studies have categorised high-grade serous ovarian cancer into subtypes but there is a lack of reproducibility between studies [9]. Tothill et al. [10] reported four HGSOC subtypes: (i) immunoreactive (ii) low stromal response (iii) high stromal response and (iv) mesenchymal. The high-stromal response and mesenchymal subtypes showed poorer survival compared with other subtypes [10]. The Cancer Genome Atlas (TCGA) project also identified four subtypes by gene expression (i) immunoreactive (ii) proliferative (iii) differentiated and (iv) and mesenchymal but found no differences in patient survival between these subtypes [11]. A consensus classifier for HGSOC has been proposed, with a subset of tumours examined unclassifiable based on currently proposed subtypes [9].

In 2013, a major study by Domcke et al. ranked ovarian cancer cell lines by their appropriateness to model HGSOC [12]. An analysis of Pubmed in 2021 showed that seven cell lines collectively constituted almost 90% of ovarian cancer cell line usage (ranked by highest usage: SKOV-3, A2780, OVCAR-3, IGROV-1, CAOV-3, 59M and OVCAR-8) [13] Of these, SKOV3, A2780, IGROV-1 and OVCAR8 were categorised by Domcke et al. as inappropriate to model HGSOC.

Long-term culture of cell lines may result in genetic drift where the cell lines no longer reflect the original tumours that they are supposed to model. The scientific community is in general neglectful of routine monitoring of cell lines with genetic characterisation [14]. As many ovarian cancer cell lines have been in use for decades ahead of the Domcke et al. study, SKOV-3 (1975) IGROV-1 (1985), the question is raised: What if genetic drift occurred before the landmark 2013 study? And are there clonal populations of cell models dismissed by Domcke that could model HGSOC?

In this study we examine the mutational profile, original histology and chromosomal copy number of a panel of ovarian cancer cell lines, compare our results to the findings of Domcke et al. and suggest which may be appropriate models of various subtypes of ovarian cancer.

## Methods

### Cell culture

Cell lines HOC1, HOC7 were grown in DMEM (Invitrogen, Grand Island, NY, USA # 11995) 10% FBS (Hyclone, Logan, Utah, USA #sv30014.03); IOSE80 were grown in M199:MCDB105 (Invitrogen #11150, Sigma #M6395) 5% FBS. DOV13 were grown in MEM (Invitrogen #11095) 10% FBS with NEAA. EFO27 were grown in RPMI (ATCC #41458) 20% FBS with the addition of l-glutamine, NEAA and Na Pyruvate. The remainder of cells (SKOV3, IGROV-1-MDA, IGROV-1-NKI, PA-1), were grown in RPMI-1640 10% FBS (Biosciences, Dublin, Ireland, 10270-106-Lot 41Q2130K), the following cell lines had additives 2 mM l-glutamine (A2780). No antibiotics were used in the culture of cell lines. The IGROV-1-NKI cell line was obtained from the Netherlands Cancer Institute [15] in 2008 all other cell lines were obtained from the MD Anderson Cancer Centre in 2010.

### DNA extraction

DNA extractions were performed using the Qiagen QIAamp DNA mini kit “Appendix B: Protocol for Cultured cells” spin column protocol adding 0.4 mg RNaseA to each sample prior to the AL buffer step.

### Affymetrix 500K single-nucleotide polymorphism arrays

250 ng of genomic DNA was processed using GeneChip Mapping NspI or StyI Assay Kit (Affymetrix, Santa Clara, CA) as per the manufacturer’s protocol and hybridized to Affymetrix Mapping 500K NspI or StyI microarrays. After hybridization, array wash, stain, and scan procedures were performed per manufacturer’s protocol. Chromosomal copy number analysis was performed using a software package previously described [16].

### DNA fingerprinting

Cell lines were either authenticated by Source BioScience LifeSciences (UK) using the AmpFISTR® SGM Plus® PCR amplification kit or authenticated in the MD Anderson CCSG supported cell line characterisation core to establish identity.

### Cytotoxicity-proliferation assays

To determine the resistance to chemotherapy drugs, cells were plated into flat-bottomed, 96-well plates at the cell density of 1 × 10^3^ cells/well and allowed to attach overnight. Olaparib (AZD2281) and veliparib (ABT888) were purchased from Selleck Chemicals (Boston, MA, USA) and made up in DMSO. Cisplatin was obtained from St. James’ Hospital Pharmacy, Dublin. Wells were treated in triplicate with serial dilutions of drug in a final volume of 200 µL. Drug-free controls were included in each assay. DMSO controls were also performed for each cell line. Plates were incubated for a further 5 days at 37 °C in a humidified atmosphere with 5% CO_2_ and cell viability was determined using an acid phosphatase assay [17].

## Results

### Ovarian cell lines with few chromosomal abnormalities are likely to be type I ovarian cancer

**Fig. 1:**
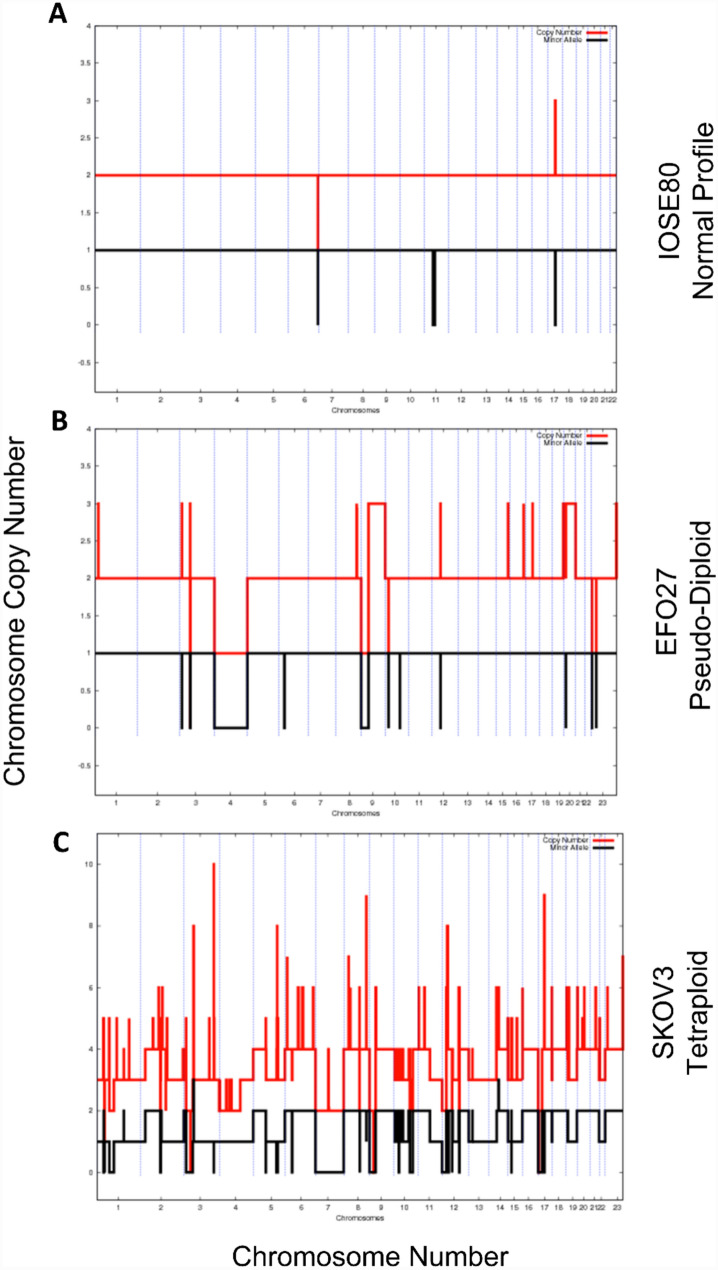
Representative chromosomal copy number profiles **A** IOSE80 with a normal diploid profile, **B** EFO27 with a pseudo-diploid profile and **C** SKOV3 with an aberrant tetraploid profile. The red line represents the copy number and the black line the minor allele. Minor Allele: the number of copies of the least frequent allele; Copy Number: the sum of the major and minor allele counts

In a previous study we profiled a large panel of 41 ovarian cancer cell lines for their *BRCA1/2* mutation and *BRCA1* methylation status [18]. A chromosomal copy number analysis was also performed which revealed that seven of the ovarian cancer cell lines had very few chromosomal abnormalities, their chromosomal profile is pseudo-diploid (A2780, DOV13, EFO27, HOC-1, HOC-7, IGROV-1, PA-1). Figure 1 presents a representative chromosomal copy number profile from (A) a normal cell line ISOE80, (B) EFO-27 with a pseudo-diploid profile and (C) SKOV3 with an aberrant tetraploid profile. The majority of models were shown to have a pseudo-diploid profile when they were originally established (Table 1), and most have a histological subtype consistent with Type I ovarian cancer. Most of the cell lines have one of the mutations associated with Type I ovarian cancer (Table 1). IGROV-1 cells have a mutational profile consistent with both Type I and Type II ovarian cancer.

**Table 1:**
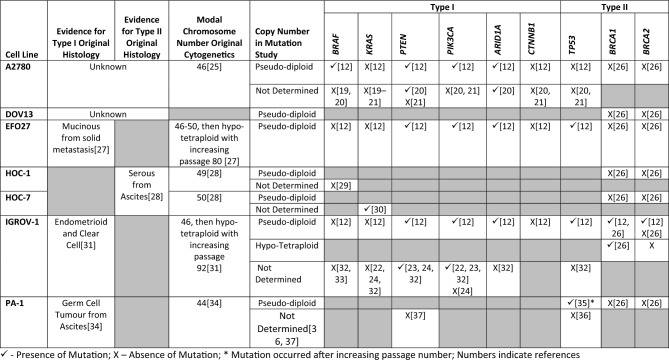
Evidence for type I vs Type II—histology and molecular markers

✓Presence of Mutation; X Absence of Mutation

*Mutation occurred after increasing passage number; Numbers indicate references

The literature disagrees about the mutational profile for several of the cell lines suggesting that there are multiple versions in use in different laboratories. A2780 has been reported to have or not have *BRAF, PTEN* and *PI3CA* mutations [12, 19–21]. IGROV-1 has been reported to have or not have *PIK3CA* and *BRCA2* mutations [12, 18, 22–24] (Table 1).

### IGROV-1 cells from different laboratories have a different chromosome profile

The IGROV-1-NKI were obtained from the Netherlands Cancer Institute in 2008 and are an 80.7% STR match to the NCI-60 IGROV-1 fingerprint (Table 2) [37]. The IGROV-1 cells were originally isolated in 1985 at the Gustave Rousey Institute (IGROV-1-GR), there is no STR fingerprint published earlier than the NCI-60 one in 2009 [30]. The IGROV-1-MDA cells were obtained from the MD Anderson Cancer Centre in 2010 and are an 84.6% match to the NCI Fingerprint (Table 2). As a guide, STR matches above 80% are considered a match, allowing a difference of one STR at one locus [37]. The IGROV-1-MDA and IGROV-1-NKI cells are a 78.1% match to each other (Table 2), which is below the threshold of an official STR match.

**Table 2:**
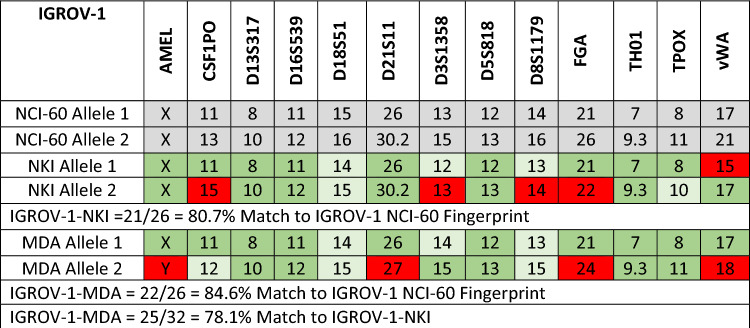
IGROV-1-NKI and IGROV-1-MDA compared to NCI-60 Reference Fingerprint

Grey—IGROV-1-NCI-60 Reference Fingerprint [37]

Dark Green—Match, Light Green ± 1 Match, Red—No Match

**Fig. 2:**
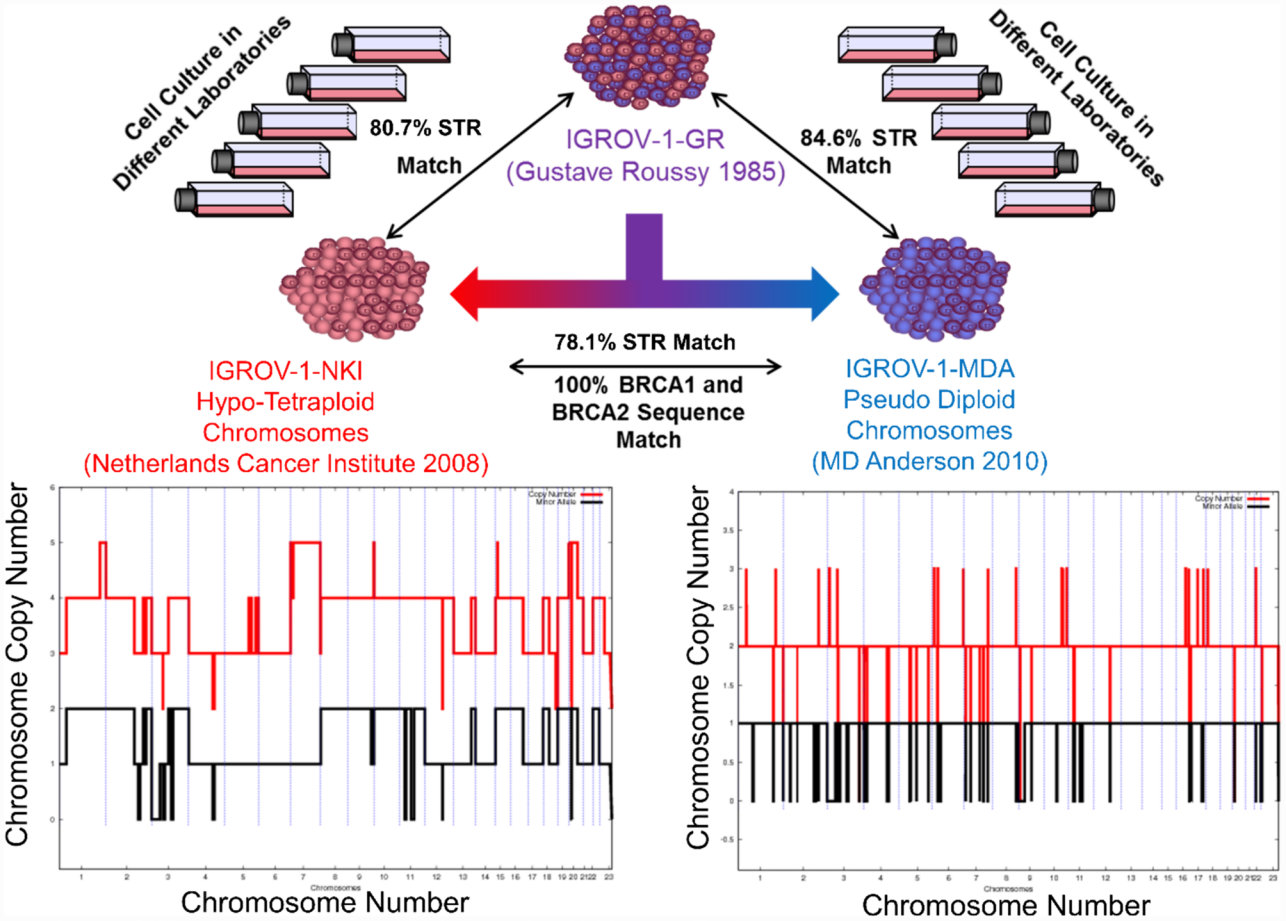
A tale of two IGROV-1s Summary – IGROV-1-NKI is an 80.7% STR match to the original IGROV-1-GR cells and has hypo-tetraploid chromosomes. IGROV-1-MDA is an 84.6% STR match to IGROV-1-GR and has pseudo-diploid chromosomes. IGROV-1-NKI and IGROV-1-MDA have a 100% match in the sequence of *BRCA1* and *BRCA2*, but are only a 78.1% STR match. The red line represents the copy number and the black line the minor allele. Minor Allele: the number of copies of the least frequent allele; Copy Number: the sum of the major and minor allele counts

The *BRCA1* and *BRCA2* gene sequences are 100% identical between IGROV-1-MDA [18] and IGROV-1-NKI, including the BRCA1 heterozygous deleterious mutation; indicating the same genetic origin (Table S1). The IGROV-1-NKI cell line has a hypo-tetraploid chromosomal profile (Fig. 2). In contrast, the IGROV-1-MDA cell line has a pseudo-normal chromosomal profile.

**Fig. 3:**
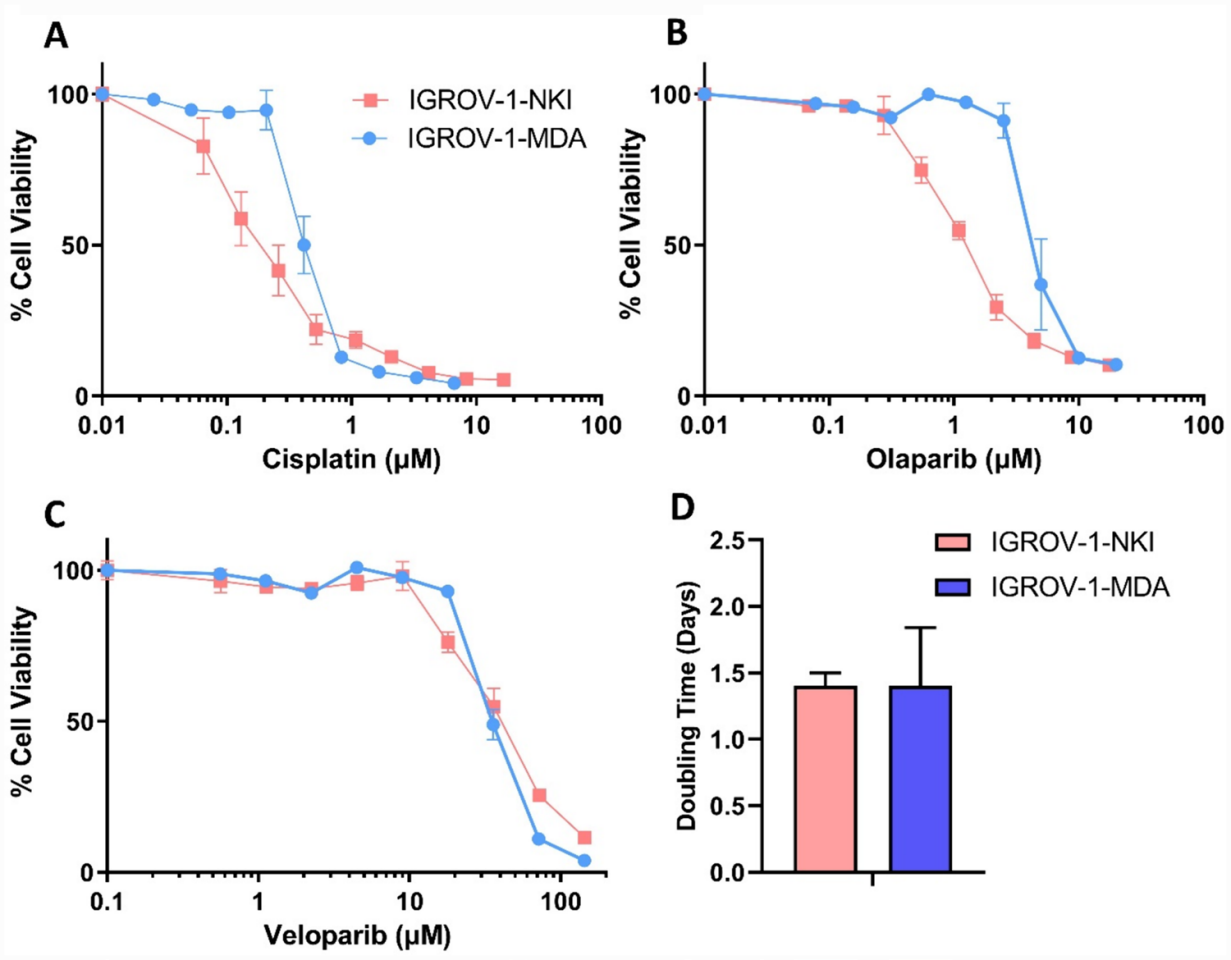
Cytotoxicity and Doubling Time in IGROV-1-NKI and IGROV-1 MDA—**A** Cisplatin, **B** Olaparib, **C** Veliparib and **D** Doubling Time. IGROV-1-NKI in pink and IGROV-1-MDA in blue. The cytotoxicity graphs are a representative replicate. The doubling time graph shows an average and standard deviation of a minimum of n = 3 replicates

The IGROV-1-NK1 and IGROV-1-MDA cell lines have a different response to chemotherapeutic drugs. The IGROV-1-MDA cells are more resistant to cisplatin and olaparib than IGROV-1-NKI (Cisplatin 0.14 ± 0.03 vs 0.31 ± 0.14 µM respectively 2.19-fold p = 0.02; Olaparib 1.24 ± 0.59 vs 6.04 ± 2.83 µM respectively 4.86-fold p = 0.0007) (Fig. 3A, B). The response to veliparib and doubling time is similar between IGROV-1-MDA and IGROV-1-NKI (Fig. 3C, D). (Veliparib 54.36 ± 9.47 vs 58.13 ± 21.59 µM respectively 1.07-fold p = 0.675; Doubling time 1.40 ± 0.1 vs 1.40 ± 0.44 days respectively 1.0-fold p = 1.0.).

## Discussion

### IGROV-1 cells have a mutational profile consistent with both Type I and Type II ovarian cancer

The original IGROV-1 study reported a mixture of cells with pseudo-diploid chromosomes and hypo-tetraploid chromosomes [30]. The hypo-tetraploid population increased with increasing passage number which would explain what we observe in the IGROV-1-NKI cells. Similarly, the original cytogenetic profiles for EFO-27 reported a mixture of cells some with a pseudo-diploid and some with an aberrant chromosome profile [26]. At high passage the pseudo-diploid population was replaced with cells with a near tetraploid number of chromosomes. What is unusual is the pseudo-diploid population being maintained in IGROV-1-MDA with the selective pressure of years of cell culture. The IGROV-1-NKI and IGROV-1-MDA cell lines have a modest difference in resistance to cisplatin and olaparib which may be related to their differing chromosomal profile.

Both the IGROV-1-NKI and IGROV-1-MDA cell lines are heterozygous for the deleterious 2080delA *BRCA1* mutation. This means that they have one functional copy of the *BRCA1* gene. We previously observed a high rate of heterozygous *BRCA1/2* mutations in ovarian cancer cell lines [18] suggesting evidence of selective pressure against cells with defects in DNA repair [38, 39]. What is interesting is that this heterozygous mutation is present in both IGROV-1-NKI and IGROV-1-MDA; suggesting that the selective pressure for the heterozygous mutation happened during the original development of the cell line and not during years of cell culture. Unfortunately, the *BRCA1/2* mutation status of the patient IGROV-1 was derived from is unknown. However, it is possible that the *BRCA1* mutation was present in the patient.

The IGROV-1 cell line was obtained from a 47-year-old woman who had stage III ovarian cancer [30]. The histological profile was described as with multiple differentiations, primarily endometrioid with some serous clear cells and undifferentiated foci [30]. This histological profile would normally be suggestive of Type I ovarian cancer and the reported mutations of *PTEN, PIK3CA* and *ARID1A* genes are consistent with this [4]. However, the *BRCA1* and *TP53* mutations suggests that it’s a Type II high-grade serous carcinoma. One explanation for these observations is that IGROV-1 is HGSOC SET (Solid, pseudo-Endometrioid and Transitional cell carcinoma-like morphology) subtype [40]. SET is common among *BRCA1*-associated ovarian cancer [40]

However, *PTEN* (3%), *PIK3CA* (3%) and *ARIDA* (3%) mutations have all been reported in Type II serous ovarian carcinomas, they are just more frequent in Type I ovarian cancers [41]. *PTEN* loss has been found in 30% of BRCA1 germline or somatic mutated ovarian tumours [42], similar to what is observed in the IGROV-1 cell line. Mutations in *ARID1A* have been reported in BRCA1 mutated ovarian cancer [43]. In the COSMIC database *PTEN* (11%), *PIK3CA* (11%) and *ARID1A* (4%) mutations all occur in BRCA1 mutated ovarian cancer [41]. IGROV-1 shares features of both Type I and Type II ovarian cancer and is modelling an unusual but previously documented group of ovarian tumours.

### Clonal populations in long-term cell culture

Scientists routinely deliberately create clonal populations of cells to study phenotypes of interest, such as chemoresistance [39, 44, 45]. However, clonal populations of cells can develop unintentionally during routine cell culture particularly if cell lines are grown for a long time.

Growing cell lines in culture is ‘survival of the fittest’ or survival of the fastest proliferating cells within the culture. Cells are subcultured because of the limited space in the culture flask. Heterogeneous tumour cell populations are diluted uniformly. As the slower growing cells are eliminated by repeated subculture, the population is selected for rapidly growing cells [46].

A study in glioblastoma found multiple clonal variants of the cell line U-251, some differing in cell surface markers. Longer-term culture of U-251 variants was associated with increased clonogenicity and tumorgenicity [14]. Comparative Genomic Hybridisation (CGH) is typically performed between a tumour cell line and a normal cell line to identify the genomic differences within the tumour. A study on MCF-7 breast cancer cells passaged in different laboratories showed substantial genetic drift between the two karyotypes by CGH [47]. MCF-7-ATCC was in culture longer than MCF-7-RIDC, and had a more complex karyotype with a higher number of chromosomes per cell (64–83 and 43–83 respectively) [47].

#### Trypsin

Trypsin is routinely used to detach attached cancer cell lines from culture flasks [48]. Cell culture protocols remind users to check for complete detachment of the cells from the flask before proceeding with sub culture [48]. There have been several reports of trypsin-resistant cell lines, which separate cells into clonal populations based on the ease at which they detach from the flask. In rat colon cancer cells, trypsin-sensitive cells that were easily detached formed tumours in syngeneic rats but were rejected within 3–4 weeks [49]. If cells are not completely detached and the same flask is used for continued culture trypsin-resistant populations may emerge. Differences in trypsinisation technique between laboratories therefore has the potential to unintentionally develop new clonal populations in long-term culture.

In this study we used a 5-min incubation with Lonza Trypsin–EDTA Mixture prepared in PBS at a working concertation of 0.25%. The original IGROV-1 study used a similar trypsin mixture but a longer exposure time of 10 min. It is unclear if this was routine practice or if the cells were hard to detach from the flask in 1985 [30]. Trypsinisation technique is not routinely reported in cell culture methods. Therefore we don’t know if there was any difference in the technique used for IGROV-1-NKI and IGROV-1-MDA [15, 18].

#### Antibiotics

With correct cell culture technique antibiotics should not be needed for the routine maintenance of cell lines [48]. A study by Elliot and Jiang found that culture of breast cancer cell lines in the antibiotic gentamicin induced gene expression of hypoxia inducer factor 1alpha, glycolytic enzymes and glucose transporters [50]. There was also an increase in reactive oxygen species causing DNA damage [50]. Human adipose-derived stem cells were also found to show different markers of differentiation and higher levels of reactive oxygen species in response to antibiotics. Long-term antibiotics use therefore has the potential to develop new subclones of a cell line.

In this study we did not use antibodies while culturing the ovarian cancer cell lines. The IGROV-1 cell line was established in primary culture using antibiotics but then maintained in antibiotic-free media [30]. The IGROV-1-NKI cells were grown in media containing antibiotics at the Netherlands Cancer Institute [15]. The IGROV-1-MDA cell line from the MD Anderson were not grown in antibiotics [18]. However, it could have been grown in antibiotics prior to our study.

### Clonal populations in in vivo models

Clonal population of cells have also been shown when cells are implanted in vivo models. Early passages of ovarian cell line EFO-27 (p12-16) consisted largely of near diploid cells with 46–50 chromosomes [26]. At p180 50% of cells had greater than 80 chromosomes, suggesting a selective pressure towards polyploidy [26]. EFO-27 cells are tumorigenic in nude mice, and cells recovered from a solid EFO-27 tumour and then cultured for 69 passages were exclusively near tetraploid [26]. This suggests that the EFO-27 cells with pseudo-diploid chromosomes are either less tumorigenic than cells with aberrant chromosomes or not tumorigenic at all.

### Relevance of the IGROV-1 model to ovarian cancer research

In 2013, a major study by Domcke et al. ranked ovarian cancer cell lines by their appropriateness to model high-grade serous ovarian cancer. IGROV-1 was ranked as a poor model and was also found to have a hyper-mutated genotype [12]. EFO27 and OC316 were also ranked as poor models with the same hyper-mutated genotype. However, the IGROV-1 cells in the Domcke et al. study were pseudo-diploid and are likely to be similar to the IGROV-1-MDA cells we have profiled. IGROV-1-NKI with its tetraploid chromosomes is likely to represent high-grade serous ovarian cancer of the SET subtype.

Many cell lines are likely to suffer from this variation across the literature. The Domcke et al. study refers to SKOV3 as having a flat pseudo-normal chromosomal profile, whereas we found an aberrant tetraploid profile (Fig. 1C). Our SKOV-3 cells were verified to have a 100% STR match to the published ATCC fingerprint [51]. The data on the SKOV3 cells in the Domcke et al. study, was derived from the Cancer Cell Line Encyclopaedia [52]. This was a large study on 947 cancer cell lines where identity was confirmed using SNP genotyping and matching to the Sanger CGP cell line project [53]. Suggesting that both cell lines were SKOV3, but different clonal populations.

## Conclusion

IGROV-1-NKI with its tetraploid chromosomes is likely to model high-grade serous ovarian cancer. Routine testing of chromosomal copy number as well as the presence of key mutations is recommended alongside STR fingerprinting to ensure that ovarian cancer cell lines are authenticated and model a specific clinical subtype.

## Supplementary Information

Below is the link to the electronic supplementary material.

Supplementary file1 (DOCX 17 KB)

## Data Availability

All data supporting the findings of this study are available within the paper and its Supplementary Information.

## Abbreviations

NKI: Netherlands Cancer Institute
MDA: MD Anderson Cancer Centre
SET: Solid, pseudo-Endometroid and Transitional cell carcinoma-like
STR: Single Tandem Repeat
