# Supplementary material for: Chromosomal copy number and mutational status are required to authenticate ovarian cancer cell lines as appropriate cell models
Source: Mol Biol Rep. 2024 Jun 28;51(1):784. doi: 10.1007/s11033-024-09747-4 (PMC11213756; doi:10.1007/s11033-024-09747-4)
Supplement: Supplementary file 1 — Supplementary file1 (DOCX 17 KB) [file 11033_2024_9747_MOESM1_ESM.docx]

**Table S1** – BRCA1/2 Sequence Data for IGROV-1-NKI and IGROV-1-MDA

| **IGROV-1-NKI BRCA1** | | | | | **IGROV-1-MDA BRCA1** [26] | | | | |
| --- | --- | --- | --- | --- | --- | --- | --- | --- | --- |
| Variant | Nucleotide Change | Exon | Predicted Protein Effect | Type of Variant | Variant | Nucleotide Change | Exon | Predicted Protein Effect | Type of Variant |
| 1 | 2080delA | 11 | Stop | Heterozygous  Deleterious | 1 | 2080delA | 11 | Stop | Heterozygous  Deleterious |
| 2 | 2201C > T | 11 | S694S | Polymorphism | 2 | 2201C > T | 11 | S694S | Polymorphism |
| 3 | 2430T > C | 11 | L771L | Polymorphism | 3 | 2430T > C | 11 | L771L | Polymorphism |
| 4 | 2731C > T | 11 | P871L | Polymorphism | 4 | 2731C > T | 11 | P871L | Polymorphism |
| 5 | 3232A > G | 11 | E1038G | Polymorphism | 5 | 3232A > G | 11 | E1038G | Polymorphism |
| 6 | 3667A > G | 11 | K1183R | Polymorphism | 6 | 3667A > G | 11 | K1183R | Polymorphism |
| 7 | 4427T > C | 13 | S1436S | Polymorphism | 7 | 4427T > C | 13 | S1436S | Polymorphism |
| 8 | 4956A > G | 16 | S1613G | Polymorphism | 8 | 4956A > G | 16 | S1613G | Polymorphism |
|  | | | | | IGROV-1-MDA = 100% Match of BRCA1 Variants to IGROV-1-NKI | | | | |
| **IGROV-1-NKI BRCA2** | | | | | **IGROV-1-MDA BRCA2** [26] | | | | |
| Variant | Nucleotide Change | Exon | Predicted Protein Effect | Type of Variant | Variant | Nucleotide Change | Exon | Predicted Protein Effect | Type of Variant |
| 1 | 5’UTR203G > A | N/A | No Effect | Polymorphism | 1 | 5’UTR203G > A | N/A | No Effect | Polymorphism |
| 2 | 3624A > G | 11 | K1132K | Polymorphism | 2 | 3624A > G | 11 | K1132K | Polymorphism |
| 3 | 4542C > T | 11 | V1438V | Polymorphism | 3 | 4542C > T | 11 | V1438V | Polymorphism |
| 4 | 7470A > G | 14 | S2414S | Polymorphism | 4 | 7470A > G | 14 | S2414S | Polymorphism |
|  | | | | | IGROV-1-MDA = 100% Match of BRCA2 Variants to IGROV-1-NKI | | | | |

Grey – IGROV-1-NKI Sequence Data; Dark Green – Match
